# Integrated transcriptomic and functional analysis reveals overlapping pathways in lung adenocarcinoma and chronic obstructive pulmonary disease

**DOI:** 10.1186/s41065-025-00625-y

**Published:** 2025-12-26

**Authors:** Dan Zhu, Jun Zhu

**Affiliations:** Department of Respiratory and Critical Care Medicine, The First Affiliated Hospital of Shaoyang University, Shaoyang, 422000 China

**Keywords:** LUAD, COPD, Hub genes, Biomarker, Treatment

## Abstract

**Background:**

Lung adenocarcinoma (LUAD) remains a leading cause of cancer-related mortality, with chronic obstructive pulmonary disease (COPD) identified as a major risk factor. However, the molecular overlap between LUAD and COPD remains poorly understood. This study aimed to identify shared hub genes and to evaluate their functional significance in LUAD.

**Methods:**

Differential gene expression analysis was conducted using two LUAD (GSE19188, GSE18842, and GSE31210) and two COPD (GSE76925, GSE57148, and GSE137557) datasets from the Gene Expression Omnibus (GEO) database. Common hub genes were identified by Venn diagram intersection of the top 3,000 DEGs per dataset. Validation was performed via RT-qPCR in LUAD (A549 and H1299) and COPD cell models. Additional transcriptomic and proteomic validations were done using GSCA, OncoDB, and HPA databases. miRNA–mRNA interactions were predicted using TargetScan and validated by TaqMan RT-qPCR. Functional assays, including CCK-8, colony formation, and wound healing, were performed after overexpression of SYNE1 and SULT1A1 in LUAD cell lines.

**Results:**

Four common hub genes, including SYNE1, SULT1A1, FAM76A, and COL10A1 were identified in both LUAD and COPD. SYNE1, SULT1A1, and FAM76A were significantly downregulated, while COL10A1 was upregulated. miRNAs targeting these genes (miR-22-3p, miR-17-3p, miR-455-3p.2, and miR-1297) were significantly upregulated in LUAD and COPD models. Immune correlation analysis revealed associations between hub gene expression and immune subtypes, immune checkpoint regulators, and drug resistance. Functional assays demonstrated that overexpression of SYNE1 and SULT1A1 suppressed proliferation, colony formation, and migration in LUAD cells. Immune correlation analysis revealed associations between hub gene expression and immune subtypes, immune checkpoint regulators, and drug resistance.

**Conclusion:**

This study identifies shared molecular signatures between LUAD and COPD.

**Supplementary Information:**

The online version contains supplementary material available at 10.1186/s41065-025-00625-y.

## Introduction

Chronic Obstructive Pulmonary Disease (COPD) and lung adenocarcinoma (LUAD) are two major respiratory diseases with considerable global health burdens [1–4]. COPD is a progressive, irreversible disorder characterized by chronic inflammation and airflow limitation, primarily triggered by long-term exposure to cigarette smoke or other harmful environmental factors [5, 6]. Globally, COPD affects over 390 million individuals and is currently the third leading cause of death, according to the World Health Organization (WHO) [7–9]. LUAD, the most prevalent histological subtype of non-small cell lung cancer (NSCLC), accounts for nearly 40–50% of all lung cancer cases and is strongly linked to smoking-related pulmonary inflammation [10, 11]. Increasing epidemiological and clinical evidence supports a close association between COPD and LUAD, suggesting that individuals with COPD are at a significantly elevated risk—approximately two- to six-fold—of developing LUAD, even after adjusting for smoking status [12–15]. This correlation implies shared molecular mechanisms such as oxidative stress, epithelial-mesenchymal transition (EMT), chronic inflammation, and dysregulation of extracellular matrix (ECM) remodeling [12, 13, 16]. However, the precise molecular pathways and overlapping gene networks that contribute to both diseases remain poorly understood [17–19].

In recent years, bioinformatics-based approaches have advanced our understanding of disease mechanisms by identifying differentially expressed genes (DEGs) and key regulatory nodes known as hub genes [20–23]. Several studies have employed transcriptomic analyses to explore the molecular basis of COPD and LUAD. For example, Zhang et al. performed weighted gene co-expression network analysis (WGCNA) using the GSE10072 and GSE76925 datasets and identified 15 hub genes including GPI, EZH2, EFNA4, CORIN, SELL, and TOP2A, several of which were validated through RT-qPCR in clinical samples and A549 cells [24]. Similarly, Li et al. utilized the GSE106899 dataset and found that FZR1, MTA1, and PKMYT1 were upregulated in LUAD tissues and in cigarette smoke-exposed A549 cells, implicating these genes in disease progression [25]. Zhang et al. focused on shared DEGs between COPD and NSCLC using multiple GEO datasets and highlighted key genes such as H2AFX, MCM2, MCM3, MCM7, POLD1, and RPA1, which are involved in DNA replication and repair pathways [26]. They reported that higher expression levels of these genes were associated with poor prognosis in LUAD patients [26]. In another study, Zhou et al. used TCGA and GTEx data to identify LUAD-related hub genes including SPP1, IL6, CDH1, PECAM1, THBS1, CAV1, and DLC1, revealing their roles in ECM dynamics, immune regulation, and angiogenesis [27].

Despite these promising findings, most studies have focused exclusively on either COPD or LUAD, with limited exploration of their molecular overlap. Moreover, many studies rely solely on in silico predictions without experimental confirmation in biologically relevant models. Therefore, there is a pressing need for a comprehensive study that combines transcriptomic data integration with experimental validation to identify and confirm common hub genes driving the pathogenesis of both COPD and LUAD [28–30]. Such findings may not only enhance our understanding of the molecular link between these two diseases but also provide novel biomarkers and therapeutic targets for early diagnosis and intervention. In this study, we sought to address this gap by performing a robust integrative analysis of publicly available gene expression datasets to identify shared hub genes between COPD and LUAD. Our integrated approach reveals new insights into the common molecular mechanisms underlying COPD and LUAD and identifies potential candidate genes for further functional and clinical studies.

## Methods

### LUAD cell lines and cell culture

Two LUAD cell lines, A549 (ATCC^®^ CCL-185™) and H1299 (ATCC^®^ CRL-5803™), and one normal human bronchial epithelial cell line, BEAS-2B (ATCC^®^ CRL-9609™), were purchased from the American Type Culture Collection (ATCC, Manassas, VA, USA). Upon receipt, cell line authentication was confirmed using short tandem repeat (STR) profiling, and all lines were routinely tested to ensure they were free of mycoplasma contamination. A549 cells were cultured in F-12 K medium (Kaighn’s Modification of Ham’s F-12) supplemented with 10% fetal bovine serum (FBS) and 1% penicillin–streptomycin. H1299 cells were maintained in RPMI-1640 medium with the same supplements. The BEAS-2B normal epithelial cells were grown in BEGM™ Bronchial Epithelial Cell Growth Medium (Lonza) supplemented as per the supplier’s protocol. All cell lines were incubated at 37 °C in a humidified atmosphere containing 5% CO₂ and subcultured at 70–80% confluence using 0.25% trypsin–EDTA, The COPD cell model was established based on the FTC (functional toxicology cell-based) approach and prior experimental experience [31, 32]. Specifically, BEAS-2B cells were exposed to 8% cigarette smoke extract (CSE) for 24 h, simulating chronic inflammatory conditions [33]. Only cells between passages 3 and 10 were used in experiments to ensure consistency and minimize phenotypic drift.

### Data acquisition and preprocessing

Publicly available transcriptomic datasets were obtained from the Gene Expression Omnibus (GEO) database (https://www.ncbi.nlm.nih.gov/geo/) [34] to identify shared hub genes between LUAD and COPD. For the discovery analysis, two LUAD datasets (GSE19188 and GSE18842) and two COPD datasets (GSE76925 and GSE57148) were selected based on sample size, data quality, and platform consistency. For independent validation, two additional GEO datasets, including GSE31210 (LUAD) and GSE137557 (COPD) were analyzed. Raw expression data were downloaded and preprocessed using the R programming environment (version 4.3.1). Background correction and normalization were performed using the limma and affy packages. Probes were annotated to official gene symbols based on the corresponding platform annotation files, and probes matching multiple genes were excluded.

### Differential gene expression analysis

Differential expression analysis between disease and control samples was conducted independently for each dataset using the limma package. Genes with an adjusted *p* value less than 0.05 and an absolute log₂ fold change of at least 1 (|log₂FC| ≥ 1) were considered significantly differentially expressed. The top 3,000 differentially expressed genes (DEGs) from each dataset were selected for further comparison.

### Identification of common hub genes

To identify shared candidate hub genes between LUAD and COPD, Venn diagram analysis was performed on the DEGs from each dataset using the VennDiagram R package. The intersection of DEGs across all four datasets yielded common hub genes.

### RNA extraction and quantitative real-time PCR (RT-qPCR)

Total RNA was extracted from cells using TRIzol™ Reagent (Invitrogen, USA), following the manufacturer’s protocol. RNA concentration and purity were assessed using a NanoDrop™ spectrophotometer (Thermo Fisher Scientific), and samples with an A260/A280 ratio between 1.8 and 2.1 were used for further analysis. RNA integrity was confirmed by electrophoresis on a 1% agarose gel.

For cDNA synthesis, 1 µg of total RNA was reverse transcribed using the PrimeScript™ RT Reagent Kit (Takara, Japan) according to the manufacturer’s instructions. Quantitative real-time PCR (RT-qPCR) was performed using SYBR™ Green Master Mix (Thermo Fisher Scientific) on ABI 7500 Fast Real-Time PCR System. The relative expression levels of target genes were normalized to GAPDH as an internal control, and fold changes were calculated using the 2^^−ΔΔCt^ method. Each sample was analyzed in triplicate, and no-template controls were included to ensure specificity. Following primers were used for the amplification purpose.

- GAPDH-F 5'-ACCCACTCCTCCACCTTTGAC-3',
- GAPDH-R 5'-CTGTTGCTGTAGCCAAATTCG-3'
- SYNE1-F: 5'-AGAGCCAAGTCCTCAACCACCT-3'
- SYNE1-R: 5'-CACCGAAGCATTTGACAGGTCAC-3'
- SULT1A1-F: 5'-GGAGTTCATGGACCACAGCATC-3'
- SULT1A1-R: 5'-CCTGCCATCTTCTCCGCATAGT-3'
- FAM76A-F: 5'-CACACCCTGTTGTGAAGTGCAC-3'
- FAM76A-R: 5'-AGGCAGGAGAATCGCTTGAACC-3'
- COL10A1-F: 5'-CGCTGAACGATACCAAATGCCC-3'
- COL10A1-R: 5'-TGGACCAGGAGTACCTTGCTCT-3'

#### Expression analysis in public transcriptomic datasets

To validate the differential expression of the identified hub genes in LUAD, independent transcriptomic datasets were analyzed. Gene expression profiles were obtained from the Gene Set Cancer Analysis (GSCA) platform (http://bioinfo.life.hust.edu.cn/GSCA/) [35] which integrates TCGA and GTEx data for comprehensive tumor versus normal comparisons. Expression levels were assessed using the LUAD dataset, and statistical significance was calculated using default GSCA settings.

#### Cross-validation using OncoDB database

Further expression validation was performed using the OncoDB database (https://oncodb.org/) [36], which compiles normalized expression data from TCGA and GTEx sources. Differential expression analysis of hub genes was conducted using the LUAD cohort.

#### Protein-level validation via the human protein atlas (HPA) database

Protein expression of the hub genes in LUAD tissues was investigated using the HPA database (https://www.proteinatlas.org/) [37]. Immunohistochemical (IHC) staining data for SYNE1, SULT1A1, FAM76A, and COL10A1 were retrieved and visually inspected. Staining intensity and distribution in LUAD tumor tissues were compared to corresponding normal lung tissues.

#### Mutational and copy number variation (CNV) analysis of hub genes

The mutational profiles and CNVs of the hub genes were analyzed using the cBioPortal for Cancer Genomics (https://www.cbioportal.org/) [38], based on the TCGA-LUAD dataset (TCGA, Firehose Legacy; https://www.cbioportal.org/study/summary?id=luad_tcga). Mutation frequency, variant classification and substitution types were assessed.

#### Prognostic and methylation analysis of hub genes

The prognostic significance of the hub genes in LUAD was assessed using the KM Plotter tool (https://kmplot.com/analysis/) [39], which integrates gene expression and survival data from TCGA and GEO cohorts. Kaplan–Meier survival curves were generated for overall survival (OS) and relapse-free survival (RFS), with patients dichotomized by median gene expression.

To evaluate potential epigenetic regulation, DNA methylation levels of the hub genes were analyzed using the OncoDB platform (https://oncodb.org/) [36]. Methylation data across promoter and gene body regions were compared between LUAD tumor samples and normal lung tissues. Differences in methylation patterns were interpreted in the context of gene expression, with particular attention to promoter hypomethylation as a possible driver of transcriptional activation.

### miRNA–mRNA network analysis

To investigate the post-transcriptional regulation of hub genes, miRNA–mRNA interactions were predicted using the TargetScan database (https://www.targetscan.org/) [40].

Expression levels of these candidate miRNAs were analyzed using the UALCAN portal (https://ualcan.path.uab.edu/) [37], which integrates TCGA expression data for cancer and normal tissue samples. Differential expression analysis was conducted between LUAD tumor tissues and normal lung controls.

To validate the dysregulation of miRNAs targeting hub genes, RT-qPCR was performed. Total RNA, including small RNAs, was extracted using the miRNeasy Mini Kit (Qiagen, Germany) following the manufacturer’s protocol. cDNA synthesis was performed using the miScript II RT Kit (Qiagen), and RT-qPCR was conducted using the miScript SYBR Green PCR Kit on ABI 7500 Fast Real-Time PCR System. For detection of individual miRNAs (hsa-miR-22-3p, hsa-miR-17-3p, hsa-miR-455-3p.2, and hsa-miR-1297), TaqMan™ MicroRNA Assays (Thermo Fisher Scientific) were used, each containing gene-specific stem-loop reverse transcription primers and TaqMan probes. TaqMan™ Universal PCR Master Mix II was used for amplification. Expression levels were normalized to U6 snRNA, which was quantified using the TaqMan™ MicroRNA Control Assay. Relative expression was calculated using the 2^^−ΔΔCt^ method. All reactions were performed in triplicate.

### Correlation of hub genes with immune subtypes and immunoregulatory molecules

To explore the immunological relevance of the hub genes in LUAD, their expression across immune subtypes and their association with immune checkpoint and co-stimulatory genes were analyzed using the TISIDB database (http://cis.hku.hk/TISIDB/) [41], an integrated repository for tumor–immune system interactions. Correlation analyses were performed to assess the relationship between hub gene expression and known immune inhibitors and immune stimulators.

### Gene enrichment, immune infiltration, and drug sensitivity analysis

Protein–protein interaction (PPI) networks were constructed using the Pathway common database (https://www.pathwaycommons.org/) [42] to assess potential interactions among the hub genes and their related partners. Functional enrichment analysis was performed using the DAVID tool (https://david.ncifcrf.gov/) [43], including Gene Ontology (GO) and Kyoto Encyclopedia of Genes and Genomes (KEGG) pathway enrichment.

Immune cell infiltration and drug sensitivity analysis was conducted using the GSCA database (http://bioinfo.life.hust.edu.cn/GSCA/) [35], which integrates gene expression with pharmacogenomic profiles. Associations between gene expression and drug response (IC50 values) were evaluated using data from GDSC and CTRP datasets. Positive correlations were interpreted as indicative of potential resistance mechanisms.

### Plasmid transfection and gene overexpression

To investigate the functional role of SYNE1 and SULT1A1 in LUAD, their overexpression was induced in A549 and H1299 cells using pcDNA3.1 expression vectors containing full-length cDNA sequences of each gene (GeneChem, China). Empty vector was used as a negative control. Transfection was carried out using Lipofectamine™ 3000 (Invitrogen, USA) following the manufacturer’s protocol. After 48 h, overexpression efficiency was confirmed by RT-qPCR and Western blot analysis.

### Western blot analysis

Cells were lysed in RIPA buffer containing protease and phosphatase inhibitors. Protein concentration was determined using the BCA Protein Assay Kit (Thermo Fisher Scientific). Equal amounts of protein (20–30 µg) were separated via SDS-PAGE, transferred to PVDF membranes (Millipore), and blocked with 5% non-fat milk. Membranes were incubated with primary antibodies against SYNE1, SULT1A1, and GAPDH (loading control) (Thermo Fisher Scientific) overnight at 4 °C, followed by HRP-conjugated secondary antibodies (Thermo Fisher Scientific). Bands were visualized using ECL detection reagent (Bio-Rad) and quantified using ImageJ software.

### Cell proliferation assay (CCK-8)

Cell proliferation was assessed using the Cell Counting Kit-8 (CCK-8, Dojindo Laboratories, Japan). Transfected cells were seeded in 96-well plates at a density of 3 × 10³ cells/well. At 0-, 24-, 48-, and 72-hours post-seeding, 10 µL of CCK-8 solution was added to each well, and absorbance at 450 nm was measured after 2 h of incubation using a microplate reader.

#### Colony formation assay

For colony formation, transfected cells were seeded into 6-well plates at 500 cells/well and cultured for 10–14 days. Colonies were fixed with 4% paraformaldehyde, stained with 0.1% crystal violet, and counted manually. Colonies with more than 50 cells were considered valid.

#### Wound healing assay

Cell migration was assessed using a wound healing assay. Transfected cells were plated in 6-well plates and grown to near confluence. A linear wound was created using a sterile 200 µL pipette tip. Cells were washed with PBS to remove debris and cultured in serum-free medium. Images of the wound area were captured at 0 and 24 h using an inverted microscope. Wound closure was quantified using ImageJ.

### Drug preparation and treatment

Austocystin D (TargetMol) was dissolved in 100% DMSO (Thermo Fisher Scientific) to prepare a 10 mM stock solution and stored at − 20 °C in the dark. Working dilutions were freshly prepared in complete culture medium immediately before use, ensuring the final DMSO concentration did not exceed 0.1% (v/v) in any treatment. Cells were treated with increasing concentrations of Austocystin D (0, 0.01, 0.03, 0.1, 0.3, 1, 3, and 10 µM) for 72 h to determine half-maximal inhibitory concentrations (IC₅₀).

#### Cell viability assay

Cell viability was measured using the PrestoBlue HS Cell Viability Reagent (Thermo Fisher Scientific, P50200) following the manufacturer’s instructions. After drug exposure, reagent was added directly to wells and incubated for 30 min at 37 °C. Fluorescence intensity was recorded at 560/590 nm using a microplate reader. Data were expressed as a percentage of vehicle control, and IC₅₀ values were derived from four-parameter logistic regression of log-transformed concentration–response curves.

#### Apoptosis assay

Apoptotic cell death was quantified using the Annexin V-FITC Apoptosis Detection Kit with propidium iodide (Thermo Fisher Scientific, V13242). Following treatment with 0.3 µM Austocystin D for 24 h, cells were harvested, washed twice with 1× binding buffer, and stained with Annexin V-FITC and PI for 15 min at room temperature in the dark. Samples were analyzed by flow cytometry, and early + late Annexin V-positive populations were considered apoptotic.

#### Cell-cycle analysis

Cell-cycle distribution was determined using the FxCycle PI/RNase Staining Solution (Thermo Fisher Scientific, F10797). After 24 h of drug exposure, cells were fixed in 70% ice-cold ethanol overnight at 4 °C, washed with PBS, and stained with FxCycle reagent for 30 min at room temperature. DNA content was analyzed by flow cytometry, and cell populations in G0/G1, S, and G2/M phases were quantified using Watson Pragmatic modeling.

### Statistical analysis

All experiments were performed in biological triplicates, and data are presented as mean ± standard deviation (SD). Statistical analyses were conducted using GraphPad Prism v9.5.1 and R v4.3.1. Comparisons between two groups were made using two-tailed unpaired Student’s t-test, while one-way ANOVA followed by Tukey’s post hoc test was used for multi-group comparisons. Correlation analyses employed Spearman’s rank correlation. Kaplan–Meier survival curves were analyzed using the log-rank test, and hazard ratios (HRs) were calculated with 95% confidence intervals. For multiple testing correction, Benjamini–Hochberg FDR adjustment was applied. Value of P* < 0.05, P** < 0.01, and P*** < 0.001 were considered statistically significant.

## Results

### Data acquisition and identification of hub genes

**Fig. 1:**
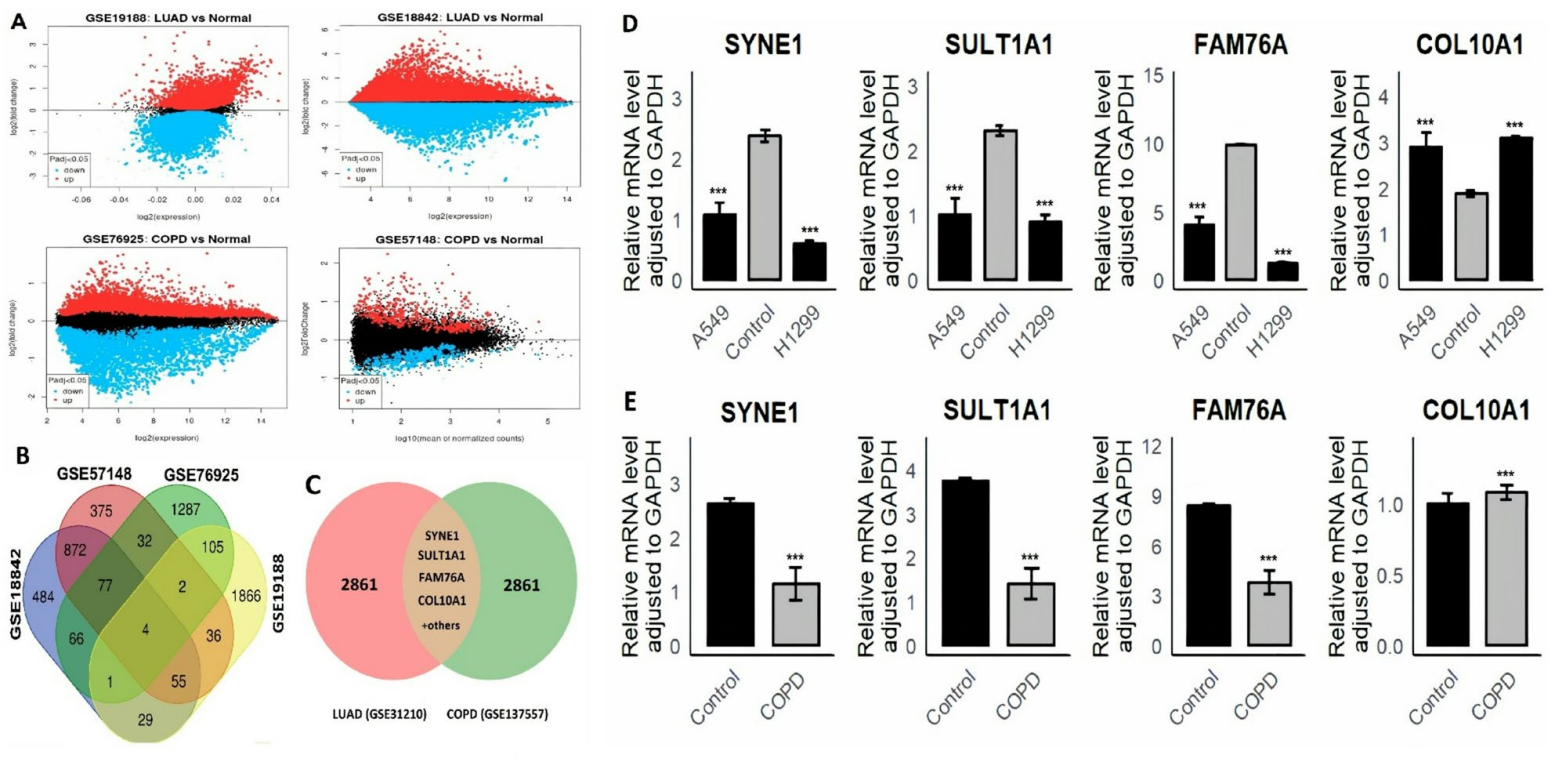
Identification and validation of common hub genes between LUAD and COPD. **A** Volcano plots showing differentially expressed genes (DEGs) in LUAD and COPD datasets. **B** Venn diagram identifying four common hub genes (SYNE1, SULT1A1, FAM76A, COL10A1) shared among top 3000 DEGs. **C** Validation Venn analysis of the top 3000 DEGs from independent datasets (GSE31210 for LUAD and GSE137557 for COPD) confirming that the same four hub genes are consistently shared between both diseases. **D** RT-qPCR analysis showing the relative mRNA expression levels of the hub genes in LUAD cell models. **E** RT-qPCR analysis showing the relative mRNA expression levels of the hub genes in LUAD cell models. P***-value < 0.001

To explore shared hub genes between LUAD and COPD, we analyzed two datasets each for LUAD (GSE19188 and GSE18842) and COPD (GSE76925 and GSE57148) from the GEO database. Differential expression analysis identified significant transcriptional changes in both diseases (Fig. 1 A). Venn analysis of the top 3000 DEGs from each dataset revealed four common hub genes: SYNE1, SULT1A1, FAM76A, and COL10A1 (Fig. 1B). To further confirm the reliability of these genes, an independent validation was carried out using two additional GEO datasets, GSE31210 for LUAD and GSE137557 for COPD. Venn analysis of the top 3000 differentially expressed genes from these validation datasets confirmed that the same four genes were consistently shared between LUAD and COPD, supporting their reproducibility and biological significance (Fig. 1 C). RT-qPCR validation showed that SYNE1, SULT1A1, and FAM76A were significantly downregulated, while COL10A1 was upregulated in LUAD and COPD cell models compared to controls (Fig. 1D–E).

### Validation of hub gene expression using additional cohorts

To further validate the expression patterns of the identified hub genes in LUAD, we utilized independent datasets. As shown in Supplementary data Fig. 1 A, analysis using the GSCA database revealed that SYNE1, SULT1A1, and FAM76A were significantly downregulated, while COL10A1 was upregulated in LUAD tumor tissues compared to normal tissues (Supplementary data Fig. 1 A). These findings were independently confirmed using the OncoDB database, where similar differential expression trends were observed across a larger LUAD patient cohort (Supplementary data Fig. 1B). At the protein level, immunohistochemical analysis from the HPA demonstrated lower expression of SYNE1, SULT1A1, and FAM76A in LUAD tissues compared to normal controls, while COL10A1 showed elevated staining intensity in tumor tissues (Supplementary data Fig. 1 C).

#### Mutational and CNV analysis of hub genes

**Fig. 2:**
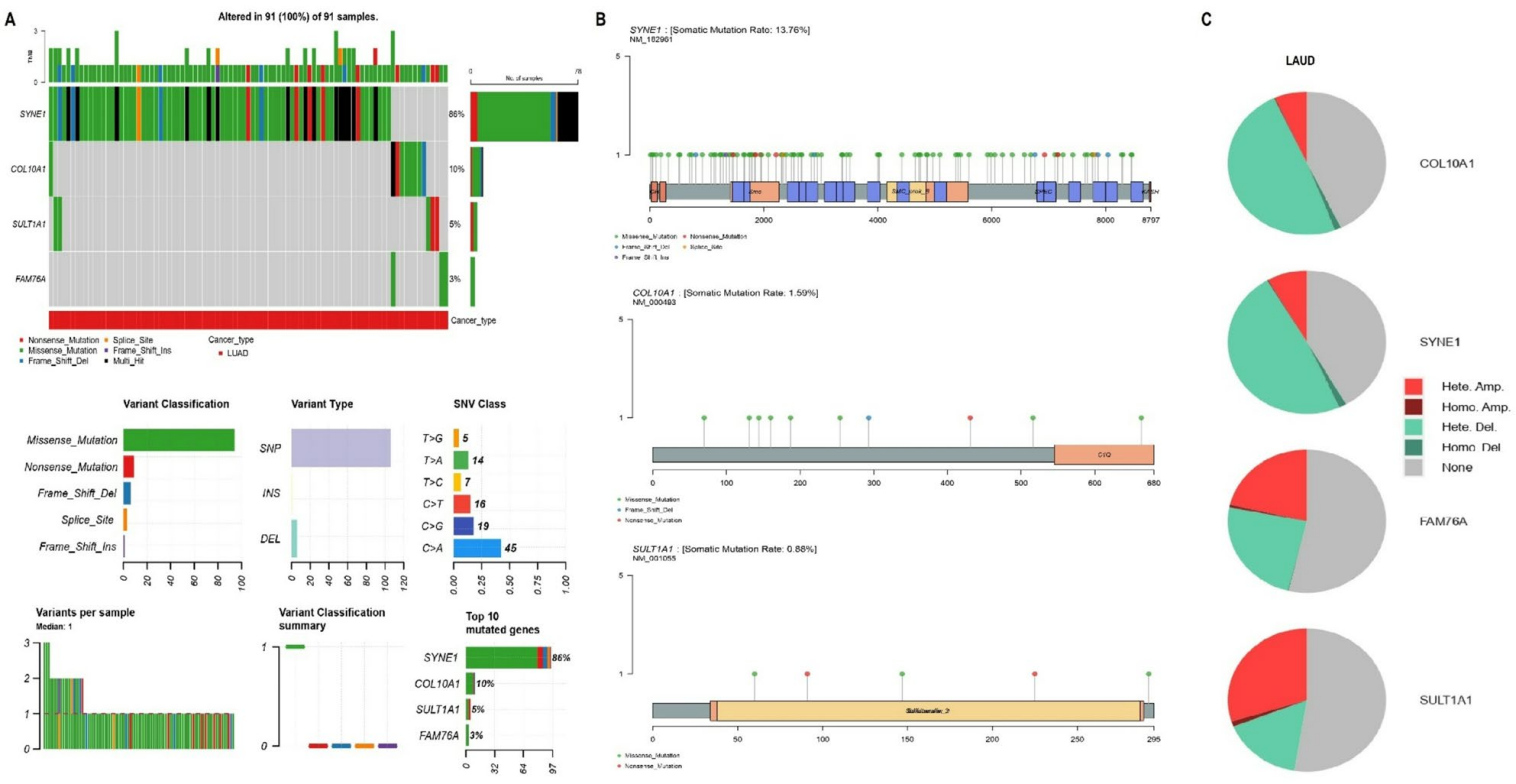
Genetic alterations and copy number variations (CNVs) of hub genes in LUAD. **A** Mutational landscape showing frequency and types of mutations in hub genes using cBioPortal.**B** Lollipop plots depicting domain-specific somatic mutations and mutation rates of hub genes.**C** CNV analysis showing frequencies of heterozygous deletions, homozygous deletions, and amplifications for each gene

**Fig. 3:**
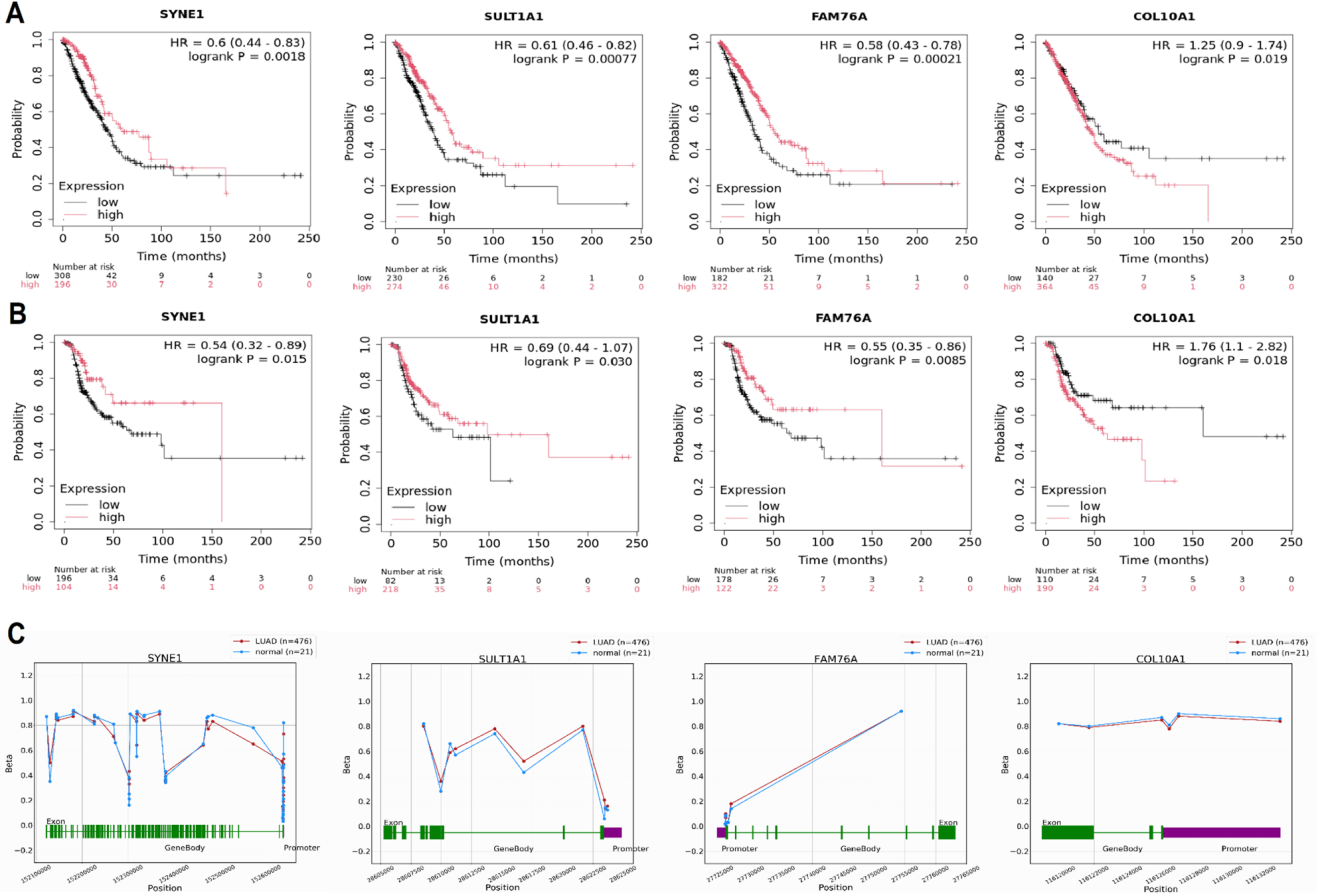
Prognostic and methylation landscape of SYNE1, SULT1A1, FAM76A, and COL10A1 in LUAD. **A** Kaplan–Meier plots for overall survival (OS) stratified by hub gene expression.**B** Relapse-free survival (RFS) analysis for high vs. low expression of hub genes.**C** DNA methylation profiles of hub genes in LUAD vs. normal tissues, focusing on promoter and gene body regions. P-value < 0.05

Mutational profiling of SYNE1, COL10A1, SULT1A1, and FAM76A in LUAD samples using cBioPortal revealed that all 91 samples (100%) harbored alterations in at least one gene (Fig. 2 A). SYNE1 showed the highest mutation frequency (86%), followed by COL10A1 (10%), SULT1A1 (5%), and FAM76A (3%) (Fig.2 A). The predominant mutation type was missense mutation, with SNPs being the most common variant type, particularly C > A transitions (Fig. 3 A). Lollipop plots (Fig. 2B) indicated domain-specific mutation clustering, with SYNE1 exhibiting a somatic mutation rate of 13.76%, COL10A1 at 1.59%, and SULT1A1 at 0.88% (Fig. 2B). CNV analysis (Fig. 2 C) revealed that heterozygous deletions were the most frequent CNV event across all genes, especially in SYNE1 and FAM76A (Fig. 2 C). Homozygous amplifications and deletions were also noted, particularly in COL10A1 and SULT1A1 (Fig. 2 C).

#### Prognostic and methylation analysis of hub genes

To evaluate the prognostic relevance of hub genes in LUAD, overall survival (OS) and relapse-free survival (RFS) analyses were performed using KM Plotter. Low expression levels of SYNE1, SULT1A1, and FAM76A were significantly associated with poor OS (HR = 0.6, 0.61, and 0.58, respectively; *P* < 0.002), while COL10A1 showed a trend toward good prognosis (HR = 1.25; *P* = 0.019) (Fig. 3 A). Consistent trends were observed in RFS analysis (Fig. 3B), where lower expression of SYNE1, FAM76A, and SULT1A1 was associated with poor outcomes, whereas COL10A1 expression correlated with reduced recurrence risk (HR = 1.76; *P* = 0.018). To explore epigenetic regulation, DNA methylation profiles of these genes were analyzed using the OncoDB database. Methylation levels across gene body and promoter regions were compared between LUAD and normal lung tissues. SYNE1, SULT1A1, and FAM76A exhibited generally lower methylation levels in LUAD samples, particularly in promoter regions, suggesting potential transcriptional activation (Fig. 3 C). In contrast, COL10A1 showed minimal methylation differences between LUAD and normal samples (Fig. 3 C).

### miRNA-mRNA network analysis

To explore the post-transcriptional regulation of hub genes, miRNA-target interactions were predicted using the TargetScan database (Supplementary data Fig. 2 A). The analysis identified four candidate miRNAs: hsa-miR-22-3p targeting SYNE1, hsa-miR-17-3p targeting SULT1A1, hsa-miR-455-3p.2 targeting FAM76A, and hsa-miR-1297 targeting COL10A1 (Supplementary data Fig. 2 A). Expression analysis of these miRNAs in LUAD tissues and normal lung samples using the UALCAN database (Supplementary data Fig. 2B) revealed significantly elevated levels in tumor samples for all four miRNAs, suggesting potential roles in tumorigenesis via downregulation of their target genes. To validate these findings, RT-qPCR was performed in LUAD cell lines (A549 and H1299) and compared with normal controls (Supplementary data Fig. 2 C). All four miRNAs were significantly upregulated in LUAD cell lines (Supplementary data Fig. 2 C). Additionally, their expression was also significantly increased in COPD cell lines compared to controls (Supplementary data Fig. 2D), indicating a potential link between chronic lung disease and LUAD-associated miRNA dysregulation.

### Correlation of hub genes with immune subtypes, immune inhibitor, and immune stimulator molecules

**Fig. 4:**
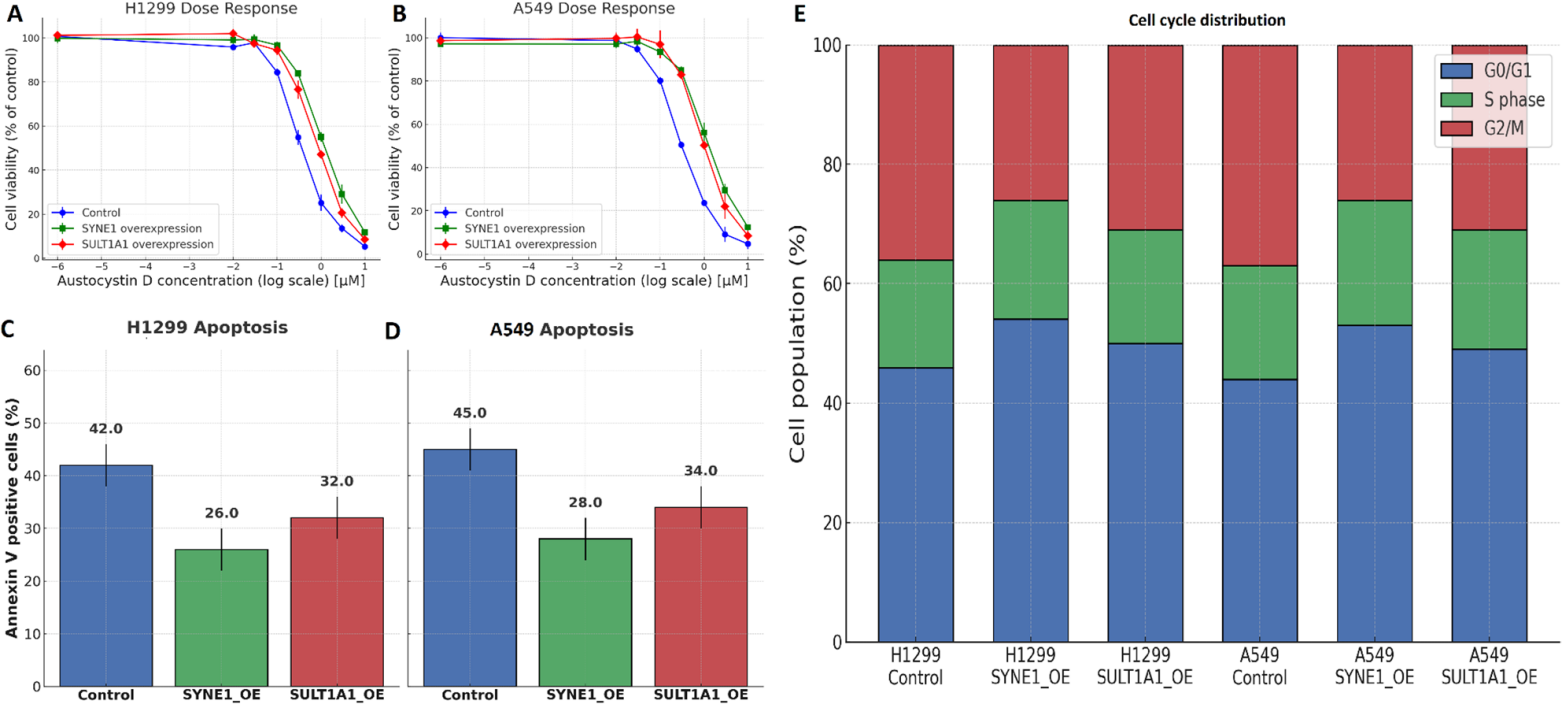
SYNE1 and SULT1A1 overexpression confer resistance to Austocystin D by attenuating apoptosis and altering cell-cycle distribution. **A**–**B** Dose–response curves showing the effect of Austocystin D on the viability of H1299 and A549 cells. SYNE1 and SULT1A1 overexpression shifted the curves to the right, indicating reduced sensitivity to the drug. **C**–**D** Quantification of apoptotic cells by Annexin V–FITC/PI flow cytometry after 24 h treatment with 0.3 µM Austocystin D. Overexpression of SYNE1 and SULT1A1 significantly decreased the proportion of Annexin V–positive cells compared with controls. **E** Cell-cycle distribution analysis following 0.3 µM Austocystin D exposure. Control cells exhibited a marked G₂/M arrest, while SYNE1- and SULT1A1-overexpressing cells showed an increased G₀/G₁ population, indicating partial escape from mitotic blockade. Data are presented as mean ± SD from three independent experiments

The expression and immune relevance of the hub genes SYNE1, SULT1A1, FAM76A, and COL10A1 were investigated across immune subtypes of LUAD using the TISIDB database. Violin plots (Supplementary data Fig. 3 A) revealed significant expression differences among six immune subtypes (C1–C6) for all four genes (Kruskal–Wallis *P* < 0.01). SYNE1 and COL10A1 showed higher expression in immune-active subtypes (C2 and C3) and lower expression in immune-suppressed subtypes (C4 and C6), suggesting their association with immunologically inflamed tumor microenvironments (Supplementary data Fig. 3 A). In contrast, SULT1A1 and FAM76A exhibited modest but distinct subtype-specific expression patterns, with relatively higher expression in C4 (Supplementary data Fig. 3 A). Correlation analysis with immune inhibitors (Fig. 4B) showed that SYNE1 and COL10A1 were positively correlated with several immune checkpoint molecules, including IL10RB, CSF1R, and CD274 (red color) (Supplementary data Fig. 3B), indicating their potential involvement in immune exhaustion (Supplementary data Fig. 3B). SULT1A1 and FAM76A showed weaker or scattered positive correlations with inhibitory genes (Supplementary data Fig. 3B). Conversely, correlation with immune stimulators (Supplementary data Fig. 3 C) revealed that SYNE1, SULT1A1, FAM76A, and FAM76A were negatively associated with key co-stimulatory molecules such as CD276 and CD28 (blue color), suggesting a potential immunosuppressive role (Supplementary data Fig. 3 C).

### Gene enrichment, immune infiltration, and drug sensitivity analysis

The functional and therapeutic relevance of the LUAD hub genes SYNE1, SULT1A1, FAM76A, and COL10A1 was further evaluated. PPI networks (Figure Supplementary data Fig. 4 A) constructed via the Pathways common database revealed extensive interactions, suggesting broad regulatory roles. Functional enrichment via DAVID showed that these genes were involved in collagen networks, ER membranes (Supplementary data Fig. 4B), oxidoreductase and monooxygenase activities (Supplementary data Fig. 4 C), and xenobiotic and alkaloid metabolism. KEGG analysis (Supplementary data Fig. 4E) highlighted enrichment in drug metabolism–cytochrome P450, chemical carcinogenesis, and steroid biosynthesis pathways (Supplementary data Fig. 4E). Immune infiltration analysis (Supplementary data Fig. 4 F) demonstrated that SYNE1 was positively correlated with immune cells such as macrophages and NK cells, indicating a pro-immune role, while FAM76A showed negative correlations with Th, suggesting immunosuppressive potential (Supplementary data Fig. 4 F). Drug sensitivity analysis (Supplementary data Fig. 4G) revealed that high SYNE1 expression was associated with resistance to multiple drugs, including austocystin D and elocalcitol (red color), highlighting its potential involvement in therapy resistance.

### Overexpression of SYNE1 and SULT1A1 confers resistance to Austocystin D by reducing apoptosis and altering cell-cycle dynamics

Austocystin D treatment significantly reduced the viability of H1299 and A549 cells in a concentration-dependent manner, whereas SYNE1 and SULT1A1 overexpression shifted the dose–response curves toward higher concentrations, indicating decreased drug sensitivity (Fig. 4A–B). The concentration of 0.3 µM was selected for further experiments as it corresponded to the approximate IC₅₀ value in control cells, producing a measurable cytotoxic effect without excessive cell loss, thereby enabling downstream mechanistic analyses. At this dose, Annexin V–FITC/PI staining showed that SYNE1- and SULT1A1-overexpressing cells exhibited markedly lower apoptotic fractions compared with controls, confirming suppression of Austocystin D–induced apoptosis (Fig. 4C–D). In parallel, cell-cycle analysis revealed that the drug induced a prominent G₂/M arrest in control cells, whereas overexpression of SYNE1 or SULT1A1 increased the G₀/G₁ population and reduced G₂/M accumulation, suggesting attenuation of drug-mediated mitotic arrest (Fig. 4E). Together, these results demonstrate that SYNE1 and SULT1A1 overexpression confers resistance to Austocystin D by limiting apoptosis and altering cell-cycle dynamics.

### SYNE1 and SULT1A1 suppresses proliferation, colony formation, and migration in LUAD cells

**Fig. 5:**
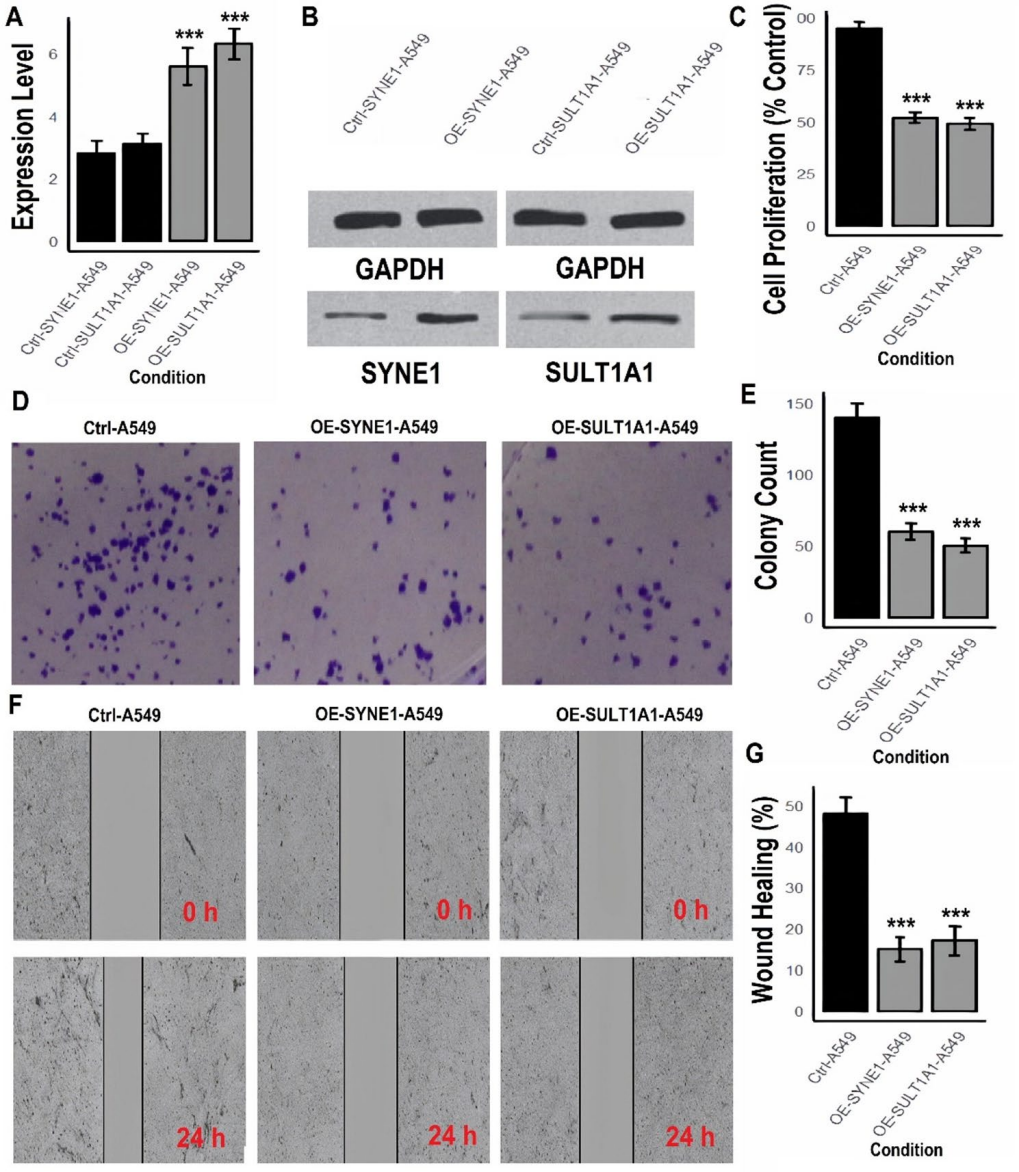
Functional assays of SYNE1 and SULT1A1 overexpression in A549 LUAD cells.**A** RT-qPCR analysis confirming SYNE1 and SULT1A1 overexpression.**B** Western blot validation of SYNE1 and SULT1A1 protein expression.**C** Proliferation assay showing reduced growth of SYNE1 and SULT1A1-overexpressing cells.**D**–**E** Colony formation assay showing reduced clonogenicity.**F**–**G** Wound healing assay showing impaired migratory capacity in SYNE1 and SULT1A1-overexpressing A549 cells. P***-value < 0.001

**Fig. 6:**
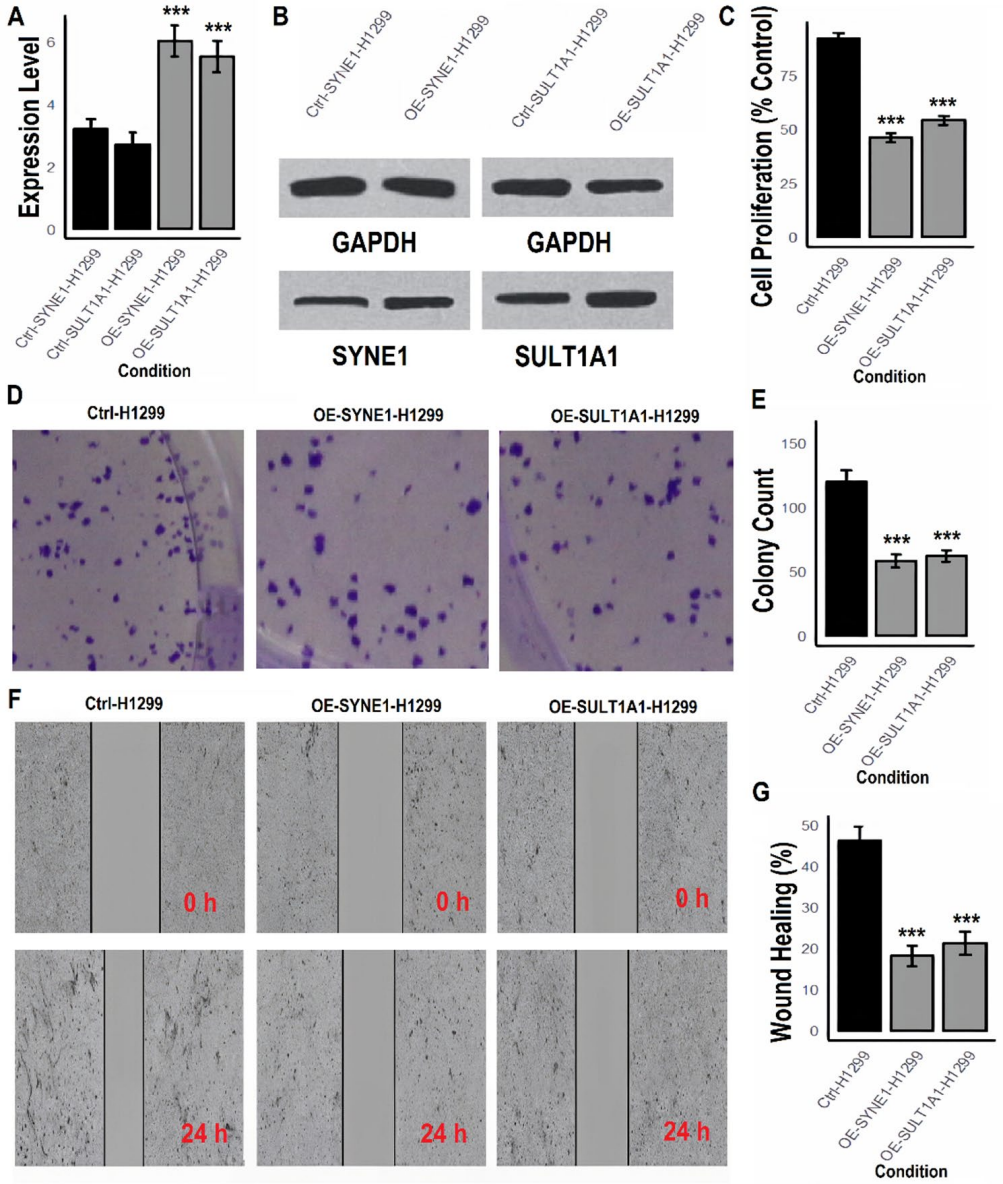
Functional assays of SYNE1 and SULT1A1 overexpression in H1299 LUAD cells. **A** RT-qPCR validation of SYNE1 and SULT1A1 overexpression.**B** Western blot confirmation of protein overexpression.**C** Cell proliferation assay indicating suppressed growth in SYNE1 and SULT1A1-overexpressing cells.**D**–**E** Colony formation reduction upon SYNE1 and SULT1A1 overexpression.**F**–**G** Wound healing assays showing significantly impaired migration in H1299 cells overexpressing SYNE1 and SULT1A1. P***-value < 0.001

To investigate the functional roles of SYNE1 and SULT1A1 in lung cancer progression, we established overexpression models in A549 and H1299 cell lines. Expression levels were validated by RT-qPCR and western blotting, followed by a series of phenotypic assays to assess their impact on tumor cell behavior. RT-qPCR analysis confirmed a significant increase in mRNA expression of SYNE1 and SULT1A1 in both A549 (Fig. 5 A) and H1299 (Fig. 6 A) overexpression models compared to their respective control groups (***p < 0.001). Western blot analysis further corroborated these results at the protein level (Figs. 5B and 6B, and Supplementary data Fig. 5), confirming successful overexpression. Cell proliferation assays demonstrated that overexpression of SYNE1 and SULT1A1 significantly reduced cell proliferation in both A549 (Fig. 5 C) and H1299 (Fig. 6 C) cells. Quantitatively, proliferation dropped to nearly 55–60% of control levels in both cell lines (***p < 0.001), suggesting a strong anti-proliferative effect. Colony formation assays revealed a marked decrease in the number of colonies formed by OE-SYNE1 and OE-SULT1A1 cells compared to control groups (Figs. 5D–E and 6D–E). The quantification indicated a greater than 60% reduction in clonogenic capacity in both cell lines (***p < 0.001), further supporting a tumor-suppressive role for both genes. Wound healing assays demonstrated a pronounced suppression of migratory ability upon overexpression of SYNE1 and SULT1A1. In contrast to the control groups that exhibited significant wound closure after 24 h, the OE-SYNE1 and OE-SULT1A1 groups displayed markedly impaired migration in both A549 (Fig. 5F–G) and H1299 (Fig. 6F–G) cells (***p < 0.001).

**Fig. 7:**
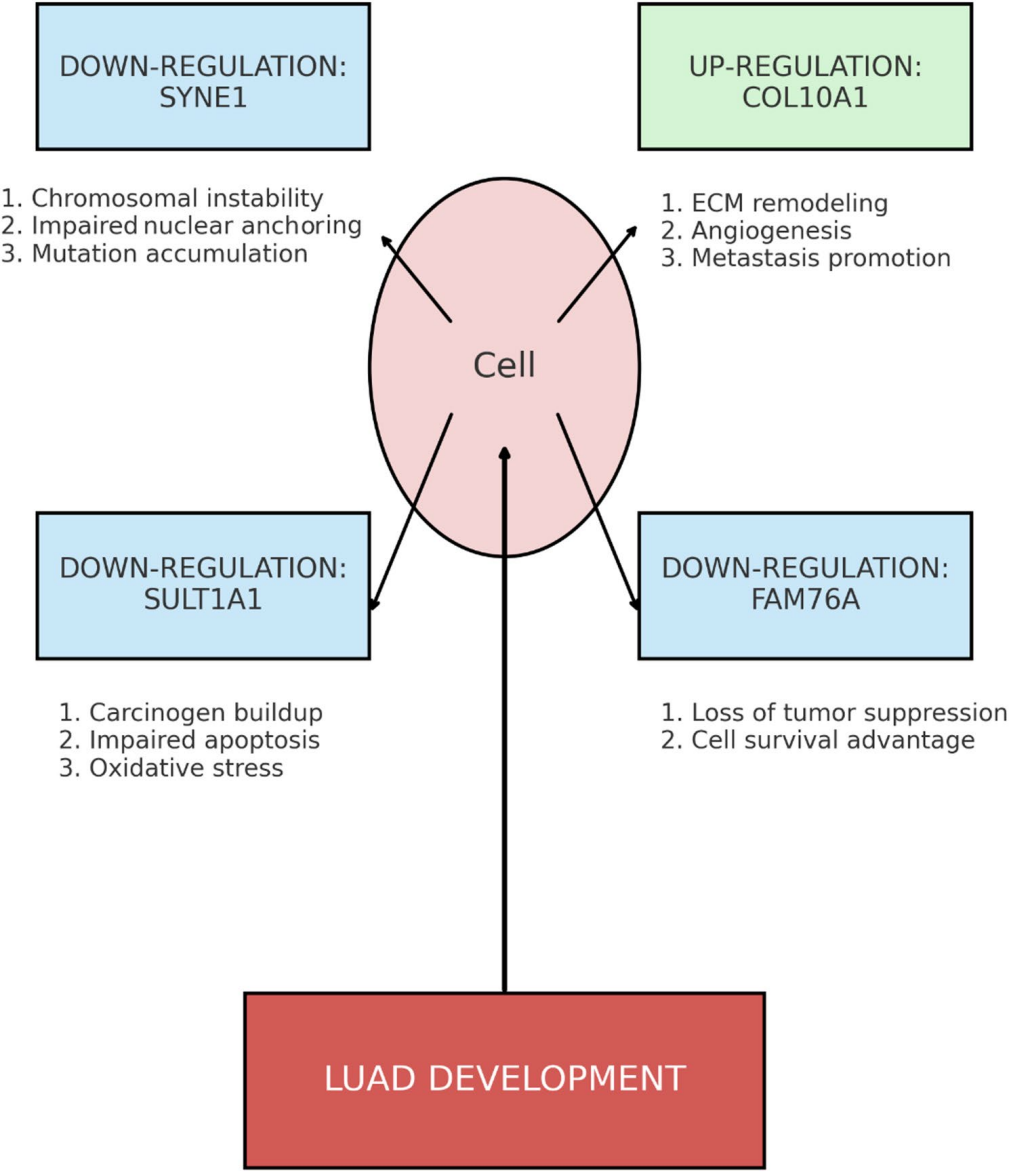
Schematic model of molecular mechanisms involving shared hub genes. Proposed pathophysiological model illustrating how SYNE1, SULT1A1, FAM76A, and COL10A1 contribute to LUAD development

Finally, in Fig. 7, the proposed pathophysiological model illustrates how dysregulation of the hub genes SYNE1, SULT1A1, COL10A1, and FAM76A potentially contributes to tumorigenesis through distinct but interconnected mechanisms. Specifically, the downregulation of SYNE1 contributes to genomic instability, aberrant cell migration, and compromised DNA repair mechanisms, all of which accelerate oncogenesis (Fig. 7). Concurrently, reduced levels of SULT1A1 hinder the detoxification of carcinogens, leading to their accumulation, increased oxidative stress, and altered cellular metabolism, thereby promoting a mutagenic environment (Fig. 7). The downregulation of FAM76A further exacerbates the situation by potentially removing a tumor suppressor function, which impairs programmed cell death (apoptosis) and allows for the uncontrolled survival and proliferation of abnormal cells (Fig. 7). Counterbalancing these suppressive losses, the upregulation of COL10A1 actively drives LUAD progression by initiating aberrant remodeling of the extracellular matrix, fostering angiogenesis for tumor nourishment, activating pro-survival and proliferative signaling pathways within cells, and facilitating epithelial-mesenchymal transition, a critical process for tumor invasion and metastasis (Fig. 7). Together, these coordinated genetic shifts create a highly permissive environment for the initiation, growth, and spread of LUAD.

## Discussion

In this study, we systematically identified four hub genes, including SYNE1, SULT1A1, FAM76A, and COL10A1 that are consistently dysregulated in both LUAD and COPD, suggesting they may underlie common pathophysiological processes. Notably, we demonstrated that SYNE1, SULT1A1, and FAM76A are downregulated, whereas COL10A1 is upregulated in multiple independent cohorts, including GEO, GSCA, OncoDB, and HPA datasets, at both transcriptional and protein levels. SYNE1 and SULT1A1 overexpression suppressed proliferation, colony formation, and migration in A549 and H1299 cell lines, corroborating their tumor-suppressive roles.

Our findings align with prior observations that COL10A1 is overexpressed in LUAD and drives ECM remodeling [44–47] via the COL10A1–DDR2–FAK axis, enhancing proliferation, invasion, and metastasis in vitro and in vivo, and correlating with poor prognosis and lymph node metastasis [48]. Additionally, our proposed mechanistic model supports this by showing COL10A1 upregulation remodels the extracellular microenvironment and activates EMT-driven motility and invasiveness. Conversely, SYNE1 has been less frequently spotlighted in lung cancer but was previously noted to be methylated and mutated in LUAD and other carcinomas [49, 50].

Our demonstration that SYNE1 downregulation leads to nuclear LINC complex collapse, genome instability, and suppressed tumor cell growth is novel [51]. It extends earlier work linking SYNE1 mutations to increased tumor mutation burden and altered immune infiltration in other malignancies such as ovarian cancer [52].

The downregulation of SULT1A1 and FAM76A, both of which are less explored in lung oncogenesis, is significant. SULT1A1’s enzymatic role in xenobiotic detoxification suggests that its reduced expression may lead to mutagen accumulation and genomic instability—a hypothesis supported by our mutation and CNV analyses [53, 54]. FAM76A, while understudied, may contribute through similar genomic or epigenetic regulatory mechanisms, as indicated by its hypomethylation in tumors [55, 56].

Our integrative genomic analysis highlights that SYNE1 harbors frequent mutations (~ 86%), including missense SNPs and heterozygous deletions, likely contributing to tumor progression. This high mutation burden aligns with the gene gravity model, where alterations in driver genes can promote broader genomic instability [57–60]. COL10A1, SULT1A1, and FAM76A exhibited lower mutation frequencies but significant CNV events, further implicating genomic dysregulation.

Clinically, low expression of SYNE1, SULT1A1, and FAM76A correlated with unfavorable overall and relapse-free survival in LUAD, consistent with their putative tumor-suppressive functions [52, 61, 62]. Conversely, COL10A1 upregulation was associated with better survival in our cohort, although prior studies generally report its overexpression portending poorer prognosis [44]. This discrepancy may result from cohort-specific heterogeneity or differences in study endpoints, highlighting the need for further validation.

Epigenetically, SYNE1, SULT1A1, and FAM76A showed lower promoter methylation in LUAD, suggesting hypomethylation may contribute to aberrant gene expression. In contrast, COL10A1 methylation remained largely unchanged, indicating other regulatory mechanisms driving its upregulation.

Finally, our immune profiling revealed complex associations between hub gene expression and tumor-infiltrating lymphocyte subtypes and checkpoint molecules. SYNE1 and COL10A1 expression correlated with immune-active subtypes (C2/C3) and positively with inhibitory receptors like IL10RB and CD274, suggesting possible involvement in shaping tumor immunologic landscapes [63, 64]. Functional enrichment implicated these hub genes in pathways including cytochrome P450 metabolism, collagen organization, and ECM remodeling, consistent with their experimentally observed phenotypes [65, 66].

Despite the comprehensive multi-omics and functional validation presented in this study, a few limitations should be acknowledged. First, while we validated gene expression using multiple public datasets and performed in vitro functional assays, in vivo experiments are needed to fully confirm the tumor-suppressive and oncogenic roles of SYNE1, SULT1A1, FAM76A, and COL10A1 in LUAD progression. Second, although our proposed pathophysiological model suggests mechanistic links between these genes and key oncogenic processes such as EMT, DNA damage, and immune modulation, these pathways were inferred from computational predictions and indirect evidence; direct mechanistic assays such as chromatin immunoprecipitation, reporter assays, or protein interaction studies were not conducted. Third, the analysis of immune infiltration and drug sensitivity relied on correlative data from bioinformatic platforms, which may not capture the full complexity of tumor-immune interactions in the tumor microenvironment. Lastly, our study focused primarily on LUAD and COPD models, and the generalizability of these findings to other lung cancer subtypes or chronic lung diseases requires further investigation in broader and more diverse patient cohorts.

## Conclusion

Our integrative analysis of expression, mutation, methylation, immune correlation, and functional assays positions COL10A1 as a likely driver of ECM-mediated tumor invasion, while SYNE1, SULT1A1, and FAM76A appear to act as tumor suppressors whose inactivation fosters genomic instability and malignant transformation. These findings are largely consistent with prior reports, particularly for COL10A1, while offering novel insights into SYNE1 and SULT1A1. Further in-depth mechanistic studies, including in vivo models and analysis of immune checkpoint interactions, are warranted to fully elucidate their roles and assess their potential as therapeutic biomarkers or targets.

## Supplementary Information

Supplementary Material 1

## Data Availability

Any type of the data will be provided by the corresponding author.
