# Supplementary material for: Integrated transcriptomic and functional analysis reveals overlapping pathways in lung adenocarcinoma and chronic obstructive pulmonary disease
Source: Hereditas. 2025 Dec 26;163:15. doi: 10.1186/s41065-025-00625-y (PMC12849426; doi:10.1186/s41065-025-00625-y)
Supplement: Supplementary file 1 — Supplementary Material 1 [file 41065_2025_625_MOESM1_ESM.pdf]

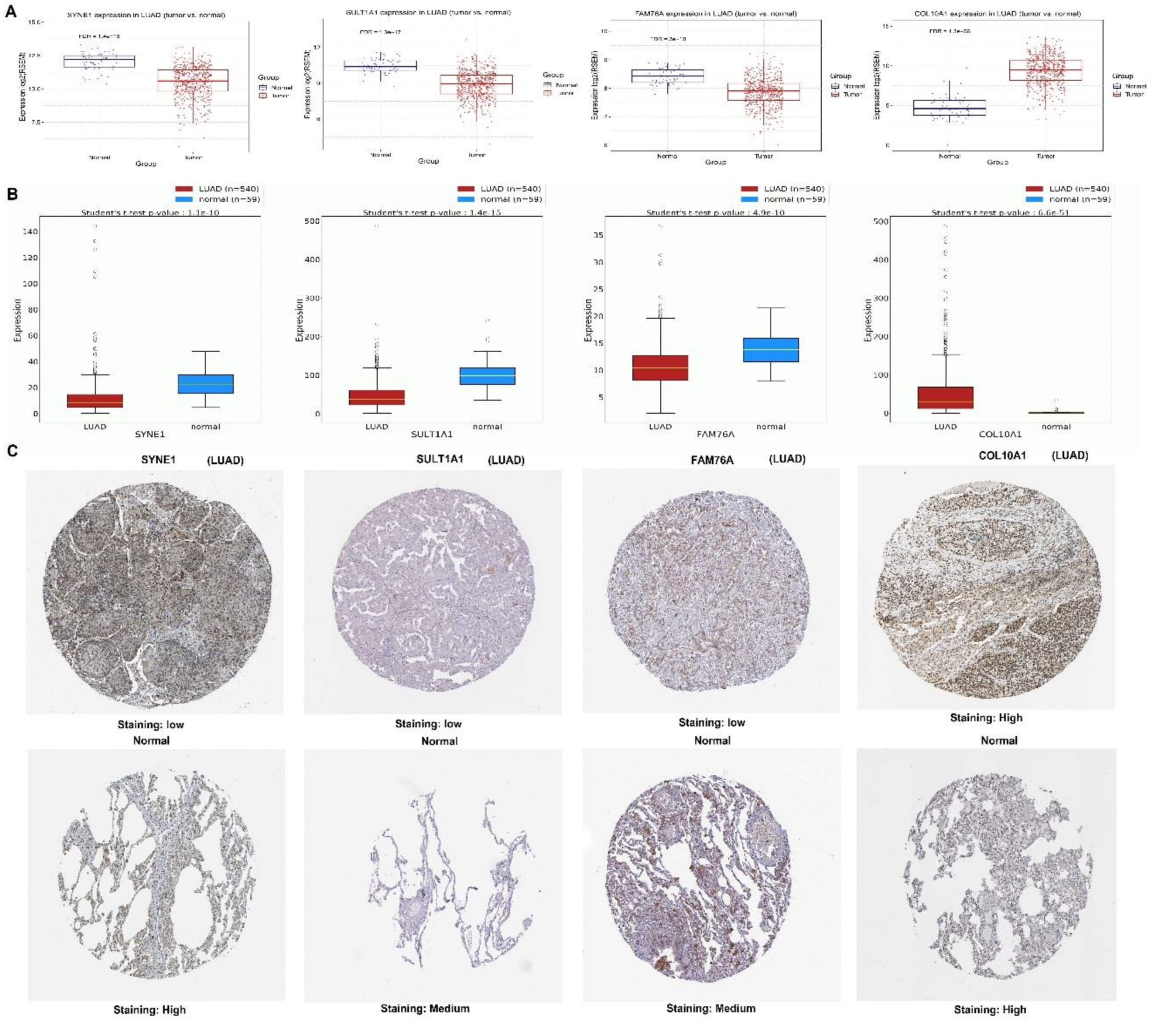

Supplementary data Figure 1. Validation of hub gene expression in LUAD tissues using external datasets. (A) Boxplots from the GSCA database showing differential expression of SYNE1, SULT1A1, FAM76A, and COL10A1 in LUAD tumor vs. normal tissues. (B) Expression validation using the OncoDB database across a large LUAD cohort. (C) Immunohistochemistry images from the Human Protein Atlas showing protein expression levels in LUAD and normal lung tissues. P-value < 0.05

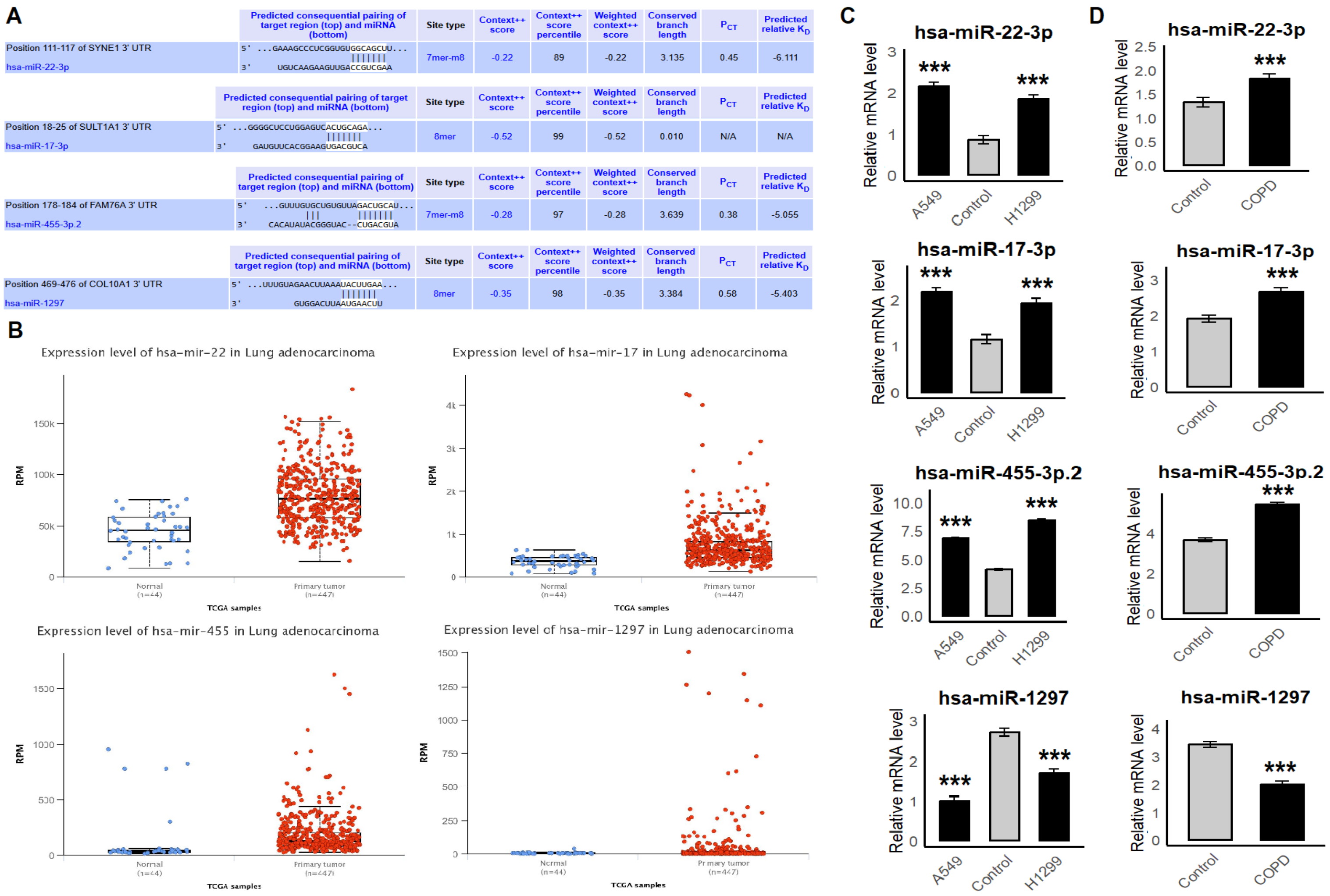

Supplementary data Figure 2. miRNA-mRNA regulatory network and validation in LUAD and COPD. (A) Predicted interactions between miRNAs and hub genes using TargetScan. (B) Expression analysis of miRNAs in LUAD tumor vs. normal tissues from UALCAN. (C) RT-qPCR validation of miRNA expression in LUAD cell lines (A549 and H1299). (D) RT-qPCR analysis of miRNAs in COPD cell lines, showing similar upregulation patterns. P***-value < 0.001

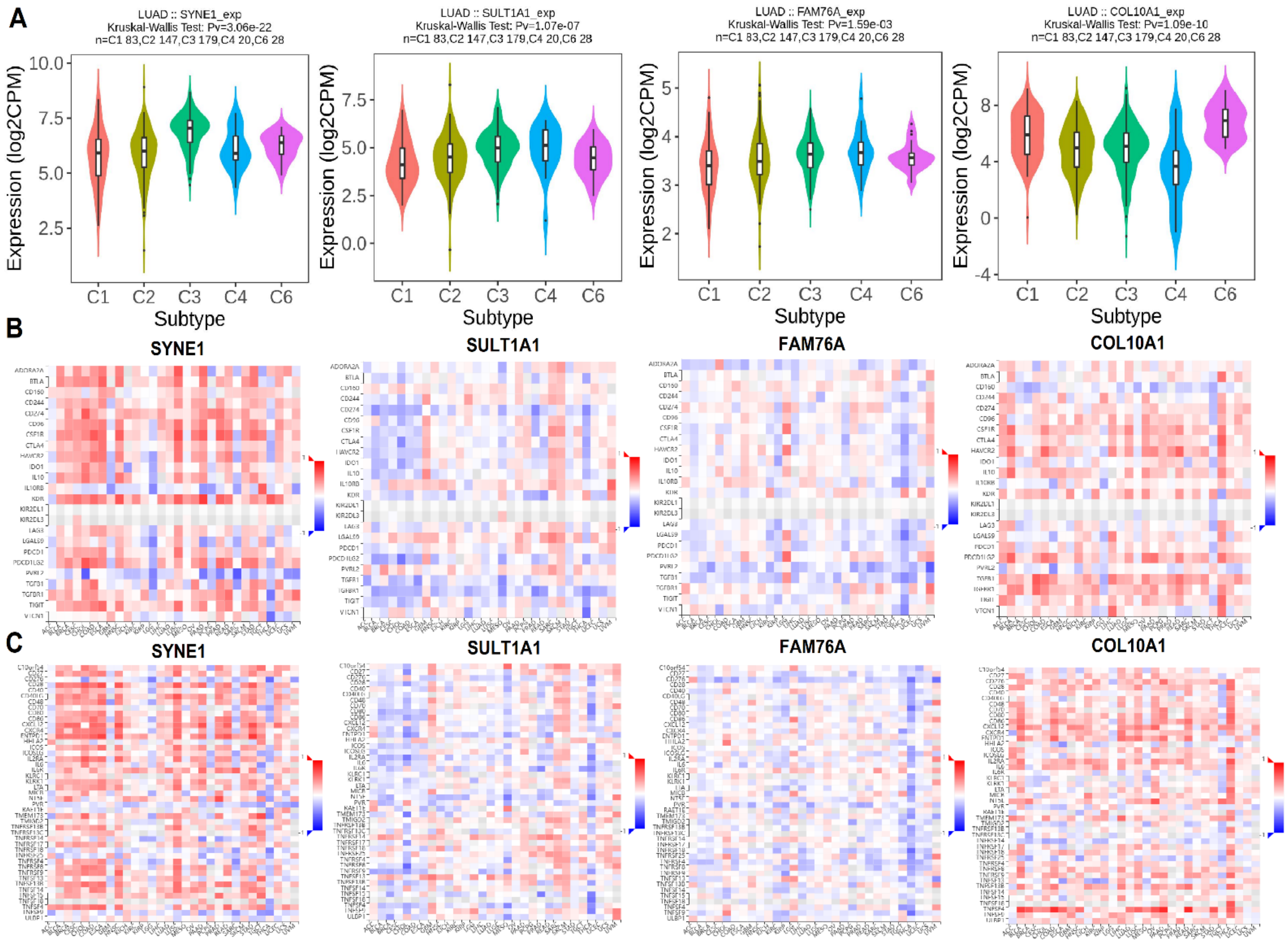

Supplementary data Figure 3. Immune subtype analysis and correlation with immune regulatory genes. (A) Violin plots showing expression of hub genes across six LUAD immune subtypes using TISIDB. (B) Correlation heatmap between hub genes and immune inhibitory checkpoint molecules. (C) Correlation heatmap between hub genes and immune stimulatory molecules. P-value < 0.05

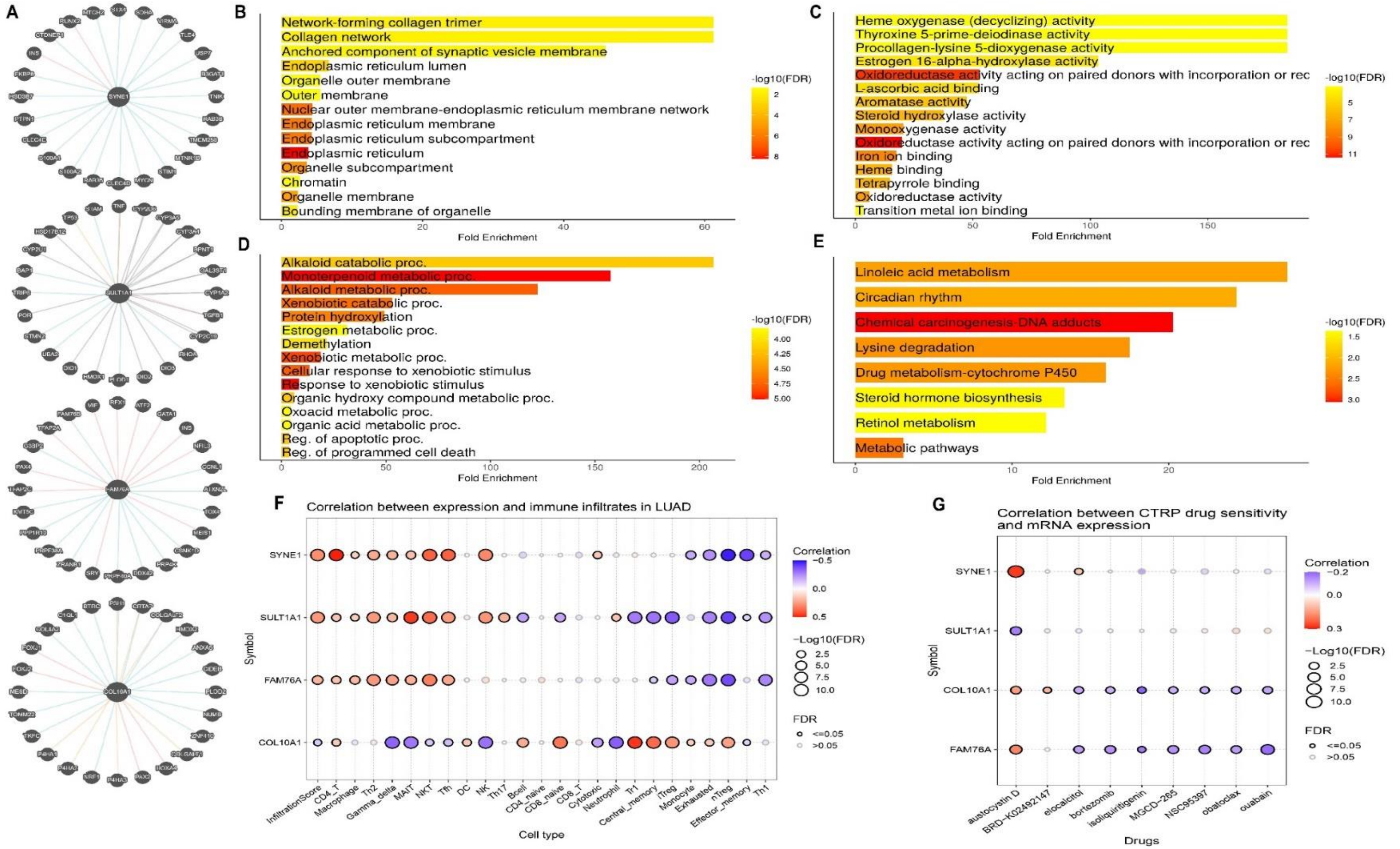

Supplementary data Figure 4. Functional enrichment, immune infiltration, and drug sensitivity of LUAD hub genes. (A) Protein–protein interaction (PPI) network of the four hub genes. (B–D) GO enrichment analysis indicating involvement in collagen structure, ER membranes, and oxidoreductase activities. (E) KEGG pathway analysis showing enrichment in cytochrome P450 drug metabolism and steroid biosynthesis. (F) Correlation of hub gene expression with immune cell infiltration in LUAD microenvironment. (G) Drug sensitivity heatmap linking hub gene expression to resistance/sensitivity to anticancer compounds. P-value < 0.05

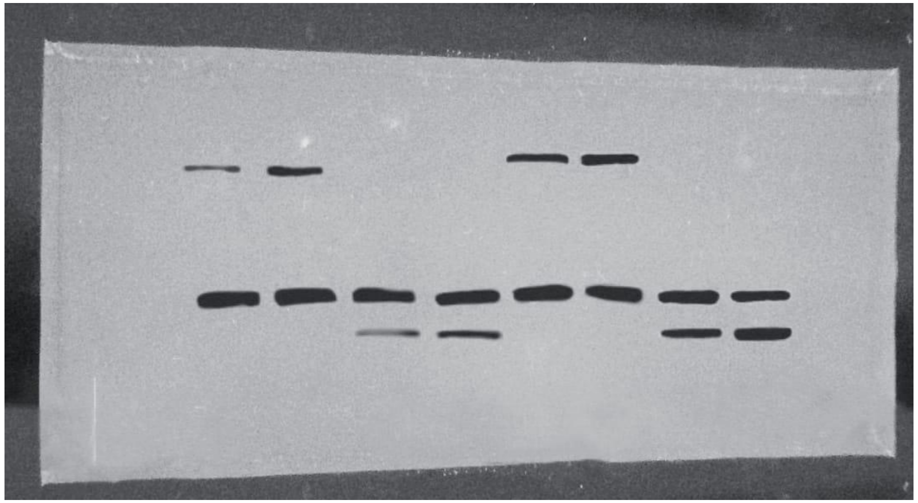

Supplementary data Figure 5: Uncut Western blot bands of SYNE1 and SULT1A1, and GAPDH.
